# Forkhead Box Protein A2 (FOXA2) Protein Stability and Activity Are Regulated by Sumoylation

**DOI:** 10.1371/journal.pone.0048019

**Published:** 2012-10-31

**Authors:** Narasimhaswamy S. Belaguli, Mao Zhang, F. Charles Brunicardi, David H. Berger

**Affiliations:** 1 Michael E. DeBakey Department of Surgery, Baylor College of Medicine, Houston, Texas, United States of America; 2 Michael E. DeBakey VA Medical Center, Baylor College of Medicine, Houston, Texas, United States of America; 3 Stem Cell Transplantation and Cellular Therapy Center, M. D. Anderson Cancer Center, Houston, Texas, United States of America; 4 David Geffen School of Medicine, University of California Los Angeles, Los Angeles, California, United States of America; North Carolina State University, United States of America

## Abstract

The forkhead box protein A2 (FOXA2) is an important regulator of glucose and lipid metabolism and organismal energy balance. Little is known about how FOXA2 protein expression and activity are regulated by post-translational modifications. We have identified that FOXA2 is post-translationally modified by covalent attachment of a small ubiquitin related modifier-1 (SUMO-1) and mapped the sumoylation site to the amino acid lysine 6 (K6). Preventing sumoylation by mutating the SUMO acceptor K6 to arginine resulted in downregulation of FOXA2 protein but not RNA expression in INS-1E insulinoma cells. K6R mutation also downregulated FOXA2 protein levels in HepG2 hepatocellular carcinoma cells, HCT116 colon cancer cells and LNCaP and DU145 prostate cancer cells. Further, interfering with FOXA2 sumoylation through siRNA mediated knockdown of UBC9, an essential SUMO E2 conjugase, resulted in downregulation of FOXA2 protein levels. Stability of sumoylation deficient FOXA2K6R mutant protein was restored when SUMO-1 was fused in-frame. FOXA2 sumoylation and FOXA2 protein levels were increased by PIAS1 SUMO ligase but not a SUMO ligase activity deficient PIAS1 mutant. Although expressed at lower levels, sumoylation deficient FOXA2K6R mutant protein was detectable in the nucleus indicating that FOXA2 nuclear localization is independent of sumoylation. Sumoylation increased the transcriptional activity of FOXA2 on *Pdx-1* area I enhancer. Together, our results show that sumoylation regulates FOXA2 protein expression and activity.

## Introduction

FOXA2 is a member of FoxA subfamily of proteins. FOXA proteins contain a centrally located DNA binding forkhead box domain [1]. The forkhead box domain of FOXA2 is nearly identical to that of FOXA1 and FOXA3, the other two members of the FOXA subfamily [2]. Because of strong similarity in their forkhead box domain, FOXA proteins bind to the same consensus TATTGA(C/T)TT(A/T)G sequence as monomers [3]. Outside of the forkhead box there is little similarity among the FOXA proteins except for the conserved activation domain II and III, located in the C-terminus, and the activation domains IV and V, located in the N-terminus [4], [5]. The forkhead domain of FOXA proteins is structurally similar to linker histones, histone 1 and 5 [6]. The conserved C-terminus of FOXA proteins interact with histones H3 and H4 and displace nucleosomal histones [7]. This unique ability to remodel chromatin and recruit cell type-restricted factors has enabled FOXA proteins to work as “pioneer factors” and initiate gene expression that confers cell- and tissue-type identities [7], [8].

FOXA2 plays a pivotal role in maintaining glucose and lipid homeostasis by regulating several genes in metabolically active tissues such as, liver, pancreatic α and β cells and adipocytes [9]–[18]. Additionally, by controlling the feeding behavior by regulating gene expression in the lateral hypothalamic neurons, FOXA2 contributes to the organismal energy balance [19]. Using genetic, biochemical and bioinformatics approaches several targets for FOXA2 in these cell types have been identified. Some of the target genes include *Pdx-1* (a pancreatic master regulator) [12], [20], [21], *Sur1* and *Kir6.2* (ATP sensitive potassium channel subunits associated with insulin secretion) [11], transthyretin and alpha-1 antitrypsin [3], *PEPCK* (a rate limiting hepatic enzyme required for gluconeogenesis) [18], [22], and orexin and melanin-concentrating hormone (promoters of feeding behavior) [19].

Several studies have shown that FOXA2 expression and activity are regulated at post-transcriptional level. FOXA2 protein but not mRNA levels are regulated in insulinoma cells by microRNA (miRNA) 124a [23]. FOXA2 is post-translationally modified by phosphorylation by casein kinase I and AKT kinase. Area IV transactivation domain located at the N-terminus contains 2 phosphorylation sites for casein kinase 1 [5]. However, these phosphorylation sites are not required for FOXA2 transcriptional activity. Insulin signaling activated AKT phosphorylates FOXA2 on threonine^156^ and translocates FOXA2 to the cytoplasmic compartment in hepatocytes [16], [22]. However, regulation of FOXA2 subcellular localization by insulin-initiated signaling has remained controversial [18]. More recently, IKKα was shown to phosphorylate FOXA2 on serine^107/111^. Serine^107/111^ phosphorylation inhibited FOXA2 transcriptional activity, derepressed FOXA2 target genes and promoted liver cancer growth [24].

We sought to identify additional modifications on FOXA2 protein and the possible mechanisms by which such modifications regulate FOXA2 protein expression and activity. In this report, we show that FOXA2 protein is modified by covalent attachment of SUMO-1 and mapped the sumoylation site to the amino acid lysine 6 (K6). By abolishing FOXA2 sumoylation or by interfering with sumoylation pathway, we show that sumoylation is required for FOXA2 protein expression. Further, we show that the stability of an inherently unstable FOXA2K6R mutant can be restored by fusing SUMO-1 in frame. Additionally, we have identified that PIAS1 is a SUMO E3 ligase for FOXA2. Finally, we demonstrate that sumoylation enhances FOXA2 transcriptional activity of *Pdx-1* area I enhancer region.

## Experimental Procedures

### Plasmids

CMV promoter-enhancer driven, HA epitope tagged Foxa2 expression vector, pCGNFoxa2, was constructed by cloning PCR amplified Foxa2 from INS-1E rat insulinoma cells into XbaI-BamHI sites of pCGN vector [25]. pCGNFoxa2K6R, in which the SUMO acceptor K6 is mutated to arginine (R) was constructed similar to pCGNFoxa2 by using a primer engineered to contain the K6R mutation. pCGNSUMO-Foxa2 was constructed by cloning PCR amplified, terminal diglycine and stop codon deleted SUMO-1 (SUMO-1ΔGGTAG) as XbaI fragment into XbaI digested pCGNFoxa2. pCGNSUMO1xFoxa2K6R was constructed similarly by cloning SUMO-1ΔGGTAG into XbaI digested pCGNFoxa2K6R. Two and 3 copy tandem repeats of SUMO-1ΔGGTAG fused to Foxa2K6R were identified by sequencing. pXFSUMO-1, a FLAG epitope tagged SUMO-1 expression vector was a kind gift from Dr. Xin-Hua Feng (Baylor College of Medicine, Houston). The PIAS1 expression vector, pCMVPIAS1 was a kind gift from Dr. Ke Shuai (University of California, Los Angeles). SUMO ligase activity deficient PIAS1C350S construct has been described previously [26]. *Pdx-1* area I region enhancer [20] regulated luciferase reporters were constructed by cloning the PCR amplified human *Pdx-1* area I region as MluI fragment into MluI sites upstream of thymidine kinase (TK) or SV40 minimal promoter luciferase vectors (Promega Corporation). A list of primers used for the recombinant DNA work and their sequences are shown in Table S1.

### Site directed mutagenesis

QuickChange II XL mutagenesis kit (Stratagene) was used to construct pCGNFoxa2K256R and pCGNFoxa2K365R, in which the potential SUMO acceptor lysines 256 and 365 are mutated to arginine. Mutagenic primer sequences are shown in the Table S1.

### Cell culture and transfections

INS-1E rat insulinoma cells [27] (a kind gift from Dr. Claes B. Wollheim) were cultured in RPMI-1640 medium supplemented with 10% FBS, 10 mM HEPES, 1 mM sodium pyruvate, 2 mM L-glutamine, 50 µM 2-mercaptoethanol, 100 units/ml penicillin and 100 µg/ml streptomycin. CV1 cells were purchased from ATCC, and maintained in DMEM supplemented with 10% FBS, 100 units/ml penicillin and 100 µg/ml streptomycin. For immunoprecipitation (IP) and western blotting experiments, INS-1E cells were plated in 6 well plates at a density of 0.5×10^6^ cells per well and transfected with 1 µg of FoxA2 or FoxA2K6R or SUMO-1 or PIAS1 or PIAS1C350S expression vectors using Lipofectamine 2000 (Invitrogen). For luciferase reporter experiments cells were plated at a density of 1×10^5^ cells per well in 24 well plates and transfected in triplicates with 0.2 µg of luciferase reporter vector and 0.4 µg of expression vector/well. For all transfections, total amount of transfected DNA was balanced using pCDNA3 empty vector. For siRNA transfections, a negative control # 1siRNA (catalog # 4611) or Ubc9 siRNAs purchased from Ambion Inc., were transfected using DharmaFect 2 (Dharmacon Inc). Three different Ubc9 siRNAs were used individually or in combination. The sequences of top strands of Ubc9 siRNAs is as follows: 5′- GCAGAGGCCUAUACAAUUUtt -3′, 5′- GCAUCUCCAUUCUGCACCAtt -3′, 5′- GCCCUAGGAGCUAGUUUCUtt -3′.

### Immunoprecipitation and western blotting

Transfected cells were lysed in FLAG buffer (300 mM NaCl, 25 mM Tris-HCl pH 8.0, 0.5% Triton X-100) and equal amounts of lysates were resolved on 10% or 8–16% gradient polyacrylamide gels. Resolved proteins were transferred onto PVDF membranes and probed with primary and appropriate HRP-conjugated secondary antibodies and the signals were detected using ECL Plus or ECL Prime kits (GE Healthcare). The primary antibodies used were: rabbit HA (Santacruz Biotechnology, catalog # sc-7392), goat FOXA2 (Santacruz Biotechnology, catalog # sc-6554), rabbit FOXA2 (Millipore, catalog # 07-633), rabbit FLAG antibody (SIGMA, catalog # F7425), rabbit SUMO-1 antibody (Cell Signaling Technology, catalog # 4930S), rabbit UBC9 antibody (Santacruz Biotechnology, catalog # sc-10759) and rabbit PIAS1 antibody (Epitomics, catalog # 2474-1). Blots were stripped using a stripping buffer (100 mM 2-mercaptoethanol, 2% SDS, 62.5 mM Tris-HCl pH 6.7) and reprobed with an internal standard goat actin antibody (Santacruz Biotechnology, catalog # sc-1615). For all IP experiments, the lysis buffer was supplemented with 20 mM N-ethyl maleimide (an isopeptidase inhibitor). Equal amounts of lysates were immunoprecipitated using 15 µl of EZView Red anti-HA affinity gel (SIGMA, catalog # E6779) and the IPs were analyzed by western blotting with rabbit HA antibody. The IP blots were stripped and reprobed with rabbit FLAG antibody. To IP endogenous FOXA2, 1 mg of whole cell extracts prepared from INS-1E cells were pre-adsorbed and IPd with 4 µg of goat FoxA2 or non-immune goat antibodies and IPs were probed with rabbit FOXA2 antibody or rabbit SUMO-1 antibody. For inhibiting proteasomal activity MG132 (SIGMA) or lactacystin (Cayman Chemicals) were added to the media to a final concentration of 10 µM 4 hours before harvesting cells. Control cells received an equal volume of the DMSO solvent.

### *In vitro* sumoylation assay

*In vitro* sumoylation assays were performed using an *in vitro* sumoylation assay kit (Enzo Biosciences) according to manufacturer's instructions. Briefly, 2 µl of [^35^S]-L-methionine labeled FOXA2 or FOXA2K6R mutant proteins produced by *in vitro* translation using rabbit reticulocyte lysate (Promega Corporation) was incubated with recombinant SAE1/2, UBC9 in the presence or absence of SUMO-1 in *in vitro* sumoylation assay buffer for 30 minutes at 30°C. The reactions were resolved on 10% polyacrylamide gel. The gel was dried and autoradiographed.

### RNA isolation and RT-PCR analysis

Total RNA was extracted using RNeasy mini kit (Qiagen). The RNA was treated with RNase free DNase (Qiagen) on column during RNA isolation to remove any contaminating DNA. Two micrograms of total RNA was used for random hexamer primed-cDNA synthesis using pre-amplification cDNA synthesis kit (Invitrogen). The levels of HAFoxa2/K6R RNAs were analyzed by semiquantitative PCR using primers corresponding to the coding region of Foxa2 (5′-CTGGGCCCACCTCACCATCCT-3′) and β-globin mRNA processing sequence (5′-GAAAACTTTGCCCCCTCCATA-3′) from the pCGN vector. GAPDH primers (5′-TGTTCCTACCCCCAATGTGT-3′ and 5′-TGTGAGGGAGATGCTCAGTG-3′) were used for internal control.

### Immunofluorescence (IF)

Subconfluent INS-1E cells plated on cover slips in 6 well plates were transfected with 1 µg of pCGN empty vector or pCGNFoxa2 or pCGNFoxa2K6R. IF experiments were performed 36 hours following transfection as described earlier [28]. Briefly, cells were fixed in 2% paraformaldehyde, permeabilized with 0.2% Triton X-100 and stained using mouse HA antibody (Santacruz Biotechnology, catalog # sc-7392) and fluorescent Alexa Fluor 594 donkey anti-mouse secondary antibody (Invitrogen, catalog # A-21203). Cover slips were mounted in DAPI containing mounting media and images were captured using BX50 (Olympus) fluorescent microscope equipped with a CCD camera.

### Statistics

Results were expressed as mean±SE and analyzed using Student's *t* test. p<0.05 was considered statistically significant.

## Results

### FOXA2 is sumoylated

**Figure 1:**
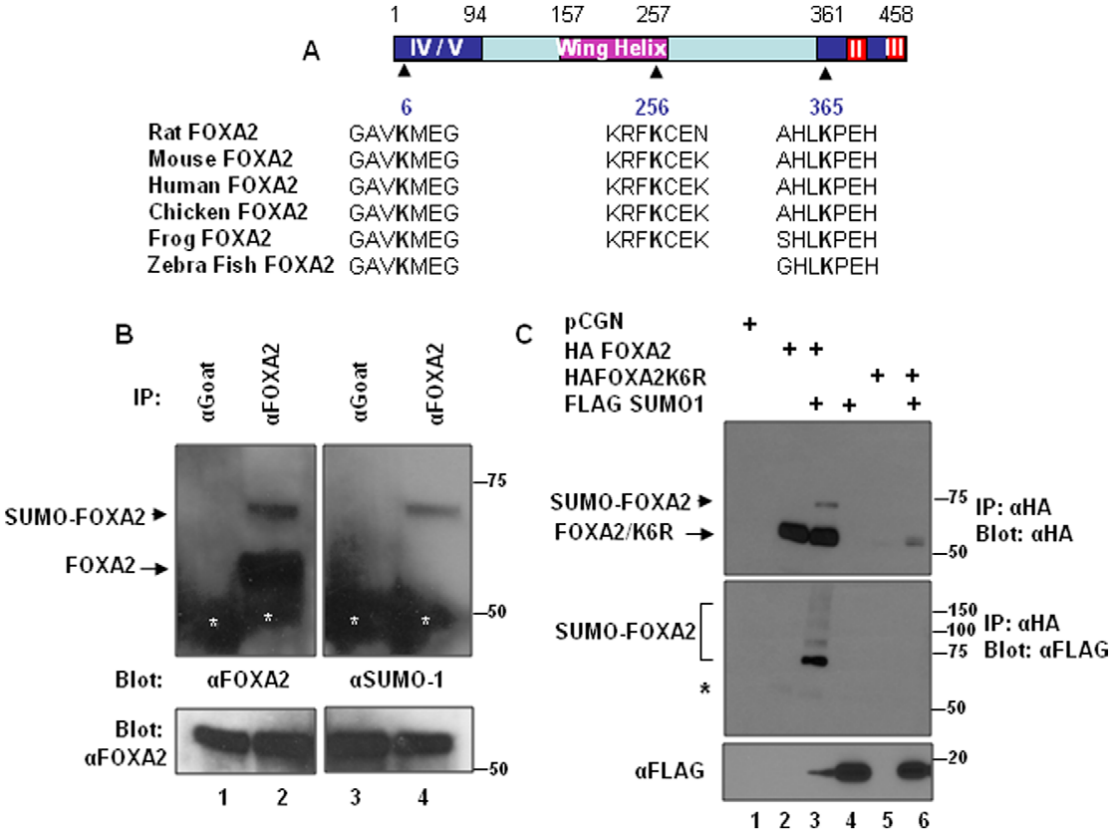
FOXA2 is sumoylated. Panel A. Rat FOXA2 protein domains are diagrammatically represented. Domain II, III, IV and V are activation domains and the winged helix domain is the DNA binding domain. Potential sumoylation sites conserved among evolutionarily distant organisms are shown below the diagram. Panel B. One mg of INS1-E whole cell extracts were immunoprecipitated with goat FOXA2 antibody (lanes 2,4) or non-immune goat antibody (lanes 1,3) and analyzed by blotting with rabbit FOXA2 antibody (left panel) or rabbit SUMO-1 antibody (right panel). Sumoylated FOXA2 and non-sumoylated FOXA2 are indicated by arrow head and arrow, respectively. White asterisks indicate IgG heavy chain. Five percent input probed with FOXA2 antibody is shown in lower panels. Panel C. INS-1E cells were transfected with indicated plasmids. Forty eight hours following transfection, 500 µg of cell lysates were immunoprecipitated with mouse HA antibody affinity gel and analyzed by western blotting with rabbit HA antibody. The blot was stripped and reprobed with FLAG antibody and shown in the lower panel. Asterisk shows signals from incompletely stripped non-sumoylated FOXA2 bands. Expression of transfected SUMO-1 is demonstrated in the bottom panel.

Sumoylation is a post-translational modification process characterized by covalent attachment of SUMO peptide to the ε amino group on lysine residues generally located in a consensus sequence ψKXE (where ψ is a hydrophobic amino acid, K is lysine, X is any residue and E is glutamic acid). Analysis of rat FOXA2 protein sequence with SUMOsp2.0, a sumoylation site prediction program (http://Sumosp.biocuckoo.org), indicated that FOXA2 has 3 potential sumoylation sites located at amino acids 6 (VKME), 256 (FKCE) and 365 (LKPE). These potential sumoylation sites are evolutionarily conserved in human, mouse, chicken, Xenopus and zebra fish FOXA2 proteins (Figure 1A). To investigate whether FOXA2 is sumoylated, we immunoprecipitated (IPd) INS-1E cell lysates with FOXA2 antibody or control goat antibody and analyzed the IPs by blotting with rabbit FOXA2 antibody or rabbit SUMO-1 antibody. FOXA2 antibody recognized 2 bands: a major 55 kD band (expected size of FOXA2) and a minor slow migrating 70 kD band (Figure 1B, left panel). The SUMO-1 antibody reacted with the minor 70 kD band in the FOXA2 IP demonstrating that this slow migrating band is the SUMO-1 modified FOXA2 (Figure 1B, right panel). To confirm that FOXA2 is sumoylated, we transfected HA epitope tagged FOXA2 along with FLAG epitope tagged SUMO-1 or the pCGN empty vector in to INS-1E cells. Immunoprecipitation (IP) with mouse HA antibody and western blotting with rabbit HA antibody revealed a 70 kD band in addition to the expected 55 kD FOXA2 band, only when SUMO-1 was cotransfected suggesting that FOXA2 is sumoylated (Figure 1C, upper panel lane 3). Upon stripping and reprobing the membrane with FLAG antibody, this 70 kD band as well as additional high molecular weight bands appeared confirming that the 70 kD band correspond to sumoylated FOXA2 (Figure 1B, lower panel lane 3).

### K6 SUMO acceptor site regulates FOXA2 protein expression

**Figure 2:**
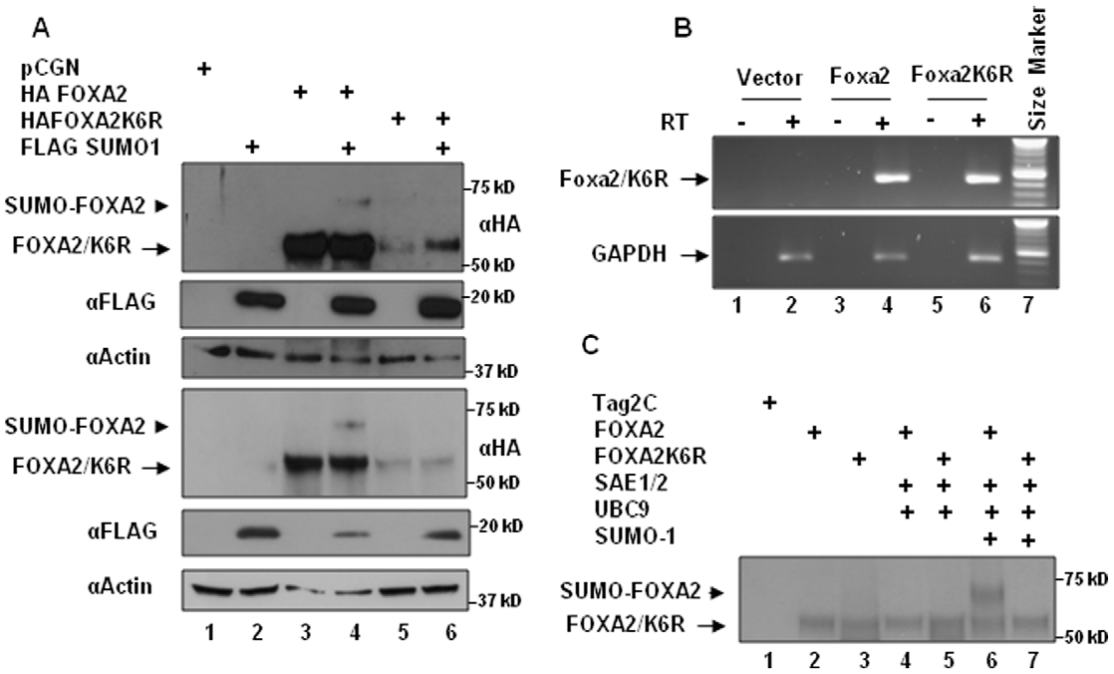
K6 SUMO acceptor site regulates FOXA2 protein expression. Panel A. Whole cell extracts prepared in the presence of 20 mM N-ethyl maleimide from INS-1E cells (panel 1) or HepG2 cells (panel 4) transfected with the indicated plasmids were analyzed by western blotting with HA antibody (panels 1, 4), FLAG antibody (panels 2, 5) and actin antibody (panels 3, 6). Sumoylated FOXA2 and non-sumoylated FOXA2/FOXA2K6R are indicated by arrow head and arrow, respectively. Panel B. cDNAs prepared in the presence (+) or absence (−) of reverse transcriptase from DNase I treated RNA from INS-1E cells transfected with pCGN vector (lanes 1,2), Foxa2 vector (lanes 3,4) or Foxa2K6R mutant vector (lanes 5,6) were analyzed by semiquantitative RT-PCR with Foxa2 primers (top panel) or GAPDH primers (bottom panel) as described in the experimental procedures. Panel C. *In vitro* sumoylation assays were performed by incubating 2 µl of [^35^S]-L-Methionine labeled FOXA2 or FOXA2K6R mutant proteins produced by *in vitro* translation with recombinant SAE1/2, UBC9 in the presence or absence of SUMO-1 in *in vitro* sumoylation assay buffer for 30 minutes at 30°C. The reactions were resolved on 10% polyacrylamide gel. The gel was dried and autoradiographed. Tag2C: rabbit reticulocyte lysate programmed with the pCMVTag2C vector.

To identify FOXA2 sumoylation site/s, we substituted K6, K256 and K365 with arginine (R) and examined these mutants for their ability to undergo sumoylation when coexpressed with FLAG-SUMO-1 in INS-1E cells. While K256R and K365R mutations did not affect sumoylation (data not shown), sumoylation was abolished by K6R mutation (Figure 2A, top panel). Additionally, the K6R mutation affected FOXA2 protein expression (top panels in figure 1C and 2A). Similar observations were made in HepG2 hepatocellular carcinoma cells (Figure 2A, 4^th^ panel) and HCT116 colon cancer cells, and LNCaP and DU145 prostate cancer cells (data not shown). Despite reduction in FOXA2K6R protein expression, Foxa2K6R mRNA levels were not affected suggesting that the K6R mutation that abolishes sumoylation also affects FOXA2 protein stability (Figure 2B). To further demonstrate that FOXA2K6 is the primary sumoylation site, we used *in vitro* sumoylation assays using bacterially expressed and purified E1, E2 and SUMO-1 and [^35^S] methionine labeled FOXA2 and FOXA2K6R proteins produced by *in vitro* translation. As shown in the figure 2C, only FOXA2 but not FOXA2K6R underwent sumoylation. Together these results demonstrate that K6 is the primary SUMO acceptor site and preventing FOXA2 sumoylation leads to destabilization and loss of FOXA2 protein expression. Unlike FOXA2 which has a short half-life of less than 2 hours [29], the half-life of FOXA2K6R could not be determined because of lower expression levels and rapid turnover.

### Interfering with sumoylation reduces FOXA2 protein levels

**Figure 3:**
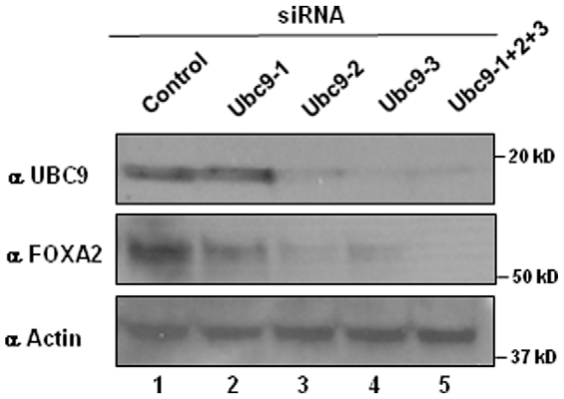
Interfering with sumoylation reduces FoxA2 protein levels. Twenty five micrograms of cell lysates from INS-1 cells transfected with control siRNA or individual or combined Ubc9 siRNAs were analyzed 48 hours post-transfection by western blotting with UBC9 antibody (top panel). Stripped blot was subsequently probed with FOXA2 antibody (middle panel) and actin antibody (lower panel).

Sumoylation is mechanistically similar to ubiquitination and involves sequential steps such as, maturation and activation of SUMO, conjugation of activated SUMO and ligation of SUMO to target proteins, catalyzed by various enzymatic activities. Unlike multiple E2 conjugating activities involved in ubiquitin conjugation, only one SUMO conjugating E2 activity (UBC9) is responsible for all sumoylations [30], [31]. To determine the role of sumoylation in FOXA2 protein expression, we knocked down UBC9 by siRNA in INS-1 cells. Knock down of endogenous UBC9 resulted in downregulation of FOXA2 protein expression suggesting that sumoylation is important for FOXA2 protein expression (Figure 3).

### Ubiquitin-proteasomal pathway contributes partially for degradation of FOXA2K6R

**Figure 4:**
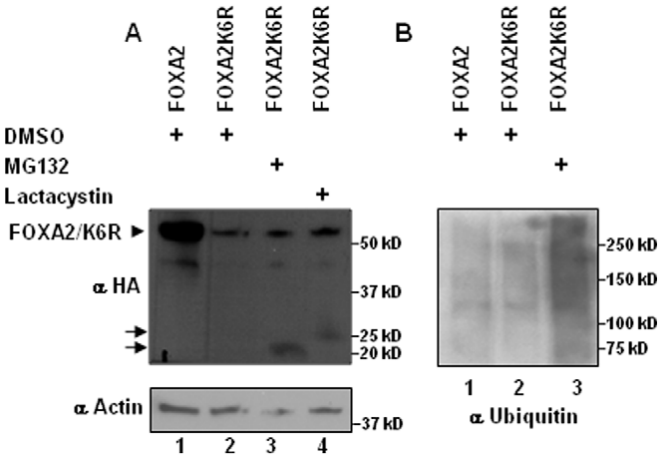
Ubiquitin-proteasomal pathway contributes partially for degradation of FOXA2K6R. Panel A. Twenty micrograms of cell lysates from INS-1E cells transfected with FOXA2 or FOXA2K6R expression vectors and treated with DMSO vehicle or MG132 or lactacystin for 4 hours were resolved on 8–16% gradient gel and analyzed by western blotting with HA antibody (top panel) or actin antibody (lower panel). Panel B. Five hundred micrograms of lysates prepared from FOXA2 or FOXA2K6R transfected cells and treated with DMSO or MG132 were immunoprecipitated with HA antibody and probed with ubiquitin antibody.

Ubiquitin proteasome system (UPS) is involved in regulated protein degradation. To investigate whether FOXA2K6R is degraded by UPS, we treated FOXA2K6R transfected cells with DMSO (vehicle) or MG132, a reversible proteasome inhibitor, and lactacystin, an irreversible proteasome inhibitor. Treatment with these proteasome inhibitors resulted in a marginal restoration of FOXA2K6R accompanied with an appearance of low molecular weight peptides (Figure 4A). Upon quantitation by NIH ImageJ and normalization to the loading control, there was a 2.3 fold and 1.8 fold increase in FOXA2K6R protein levels upon treatment with MG132 and lactacystin, respectively, compared with DMSO (vehicle) treatment. Further, western blotting analysis of HA immunoprecipitate from MG132 treated cells with ubiquitin antibody revealed a high molecular weight smear characteristic of ubiquitinated intermediates (Figure 4B). Together, these results suggest that UPS is involved, at least partially, in degrading FOXA2K6R.

### FOXA2K6R stability is restored by SUMO-1 fusion

**Figure 5:**
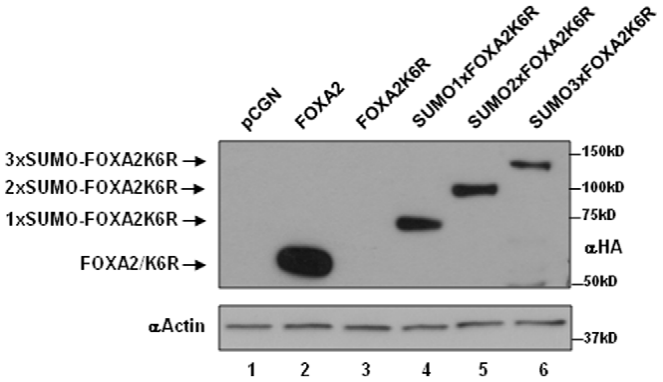
SUMO-1 fusion restores FOXA2K6R stability. Twenty micrograms of cell lysates from INS-1E cells transfected with FOXA2 or FOXA2K6R or FoxA2K6R fused to one or two or three copies of diglycine residue deleted, desumoylase resistant SUMO-1 were analyzed by western blotting with HA antibody (top panel) or actin antibody (bottom panel).

We examined whether the stability of sumoylation deficient FOXA2K6R protein can be restored by fusing SUMO-1 in frame with FOXA2K6R. To eliminate the possibility that the SUMO-1 moiety is clipped off the fusion protein by endogenous desumoylating activities (SENPs), the terminal GG-diglycine residues were deleted from SUMO-1. Fusing 1 or 2 or 3 copies of SUMO-1 to FOXA2K6R was sufficient to restore FOXA2K6R protein expression confirming that sumoylation regulates FOXA2 protein expression (Figure 5).

### FOXA2 sumoylation is promoted by PIAS1

**Figure 6:**
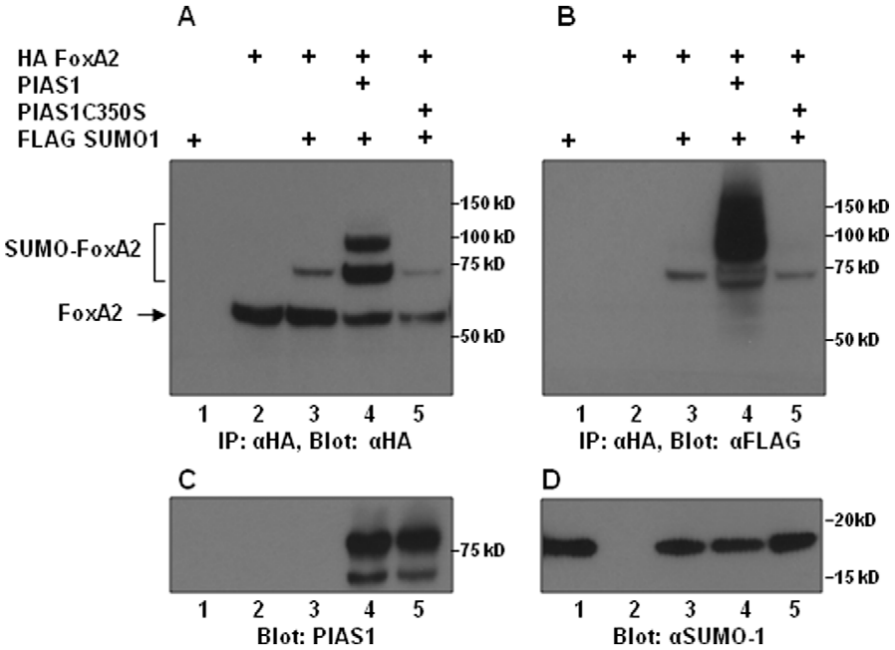
FOXA2 sumoylation is promoted by PIAS1. Five hundred micrograms of protein lysates from INS-1E cells transfected with the indicated plasmids were immunoprecipitated with mouse HA antibody and analyzed by western blotting with rabbit HA antibody (Panel A). Stripped blot probed with FLAG antibody is shown in panel B. FOXA2 and sumoylated FOXA2 are indicated by arrow and a square bracket, respectively. Expression of transfected PIAS1 and PIAS1C350S mutant and SUMO-1 are shown in panels C and D.

Unlike ubiquitination which is dependent on ubiquitin ligases, basal levels of sumoylation occurs independent of SUMO ligases [30], [31]. However, sumoylation is promoted by SUMO ligases. Therefore, we investigated whether the RING domain containing SUMO ligase, PIAS1, promotes FOXA2 sumoylation and increases FoxA2 protein steady state levels. Cotransfection of FOXA2 along with SUMO-1 resulted in readily detectable sumoylation of FOXA2 (Figure 6A, lane 3). This sumoylation was strongly enhanced when cells were cotransfected with PIAS1 expression vector but not the SUMO ligase deficient C350S mutant PIAS1 (Figure 6A, lanes 4, 5). Total amount of FOXA2 (combined sumoylated and non-sumoylated forms) was greater when FOXA2 was coexpressed with the wild-type PIAS1. In contrast, the C350S mutant of PIAS1 that acts as a dominant negative mutant of PIAS1 [32], [33], resulted in lower levels of sumoylated and non-sumoylated FOXA2 protein accumulation (Figure 6A, lane 5). Together, these results show that PIAS1 is a SUMO E3 ligase for FOXA2 and PIAS1 promoted sumoylation regulates FOXA2 protein expression.

### Nuclear localization of FOXA2 is independent of sumoylation

**Figure 7:**
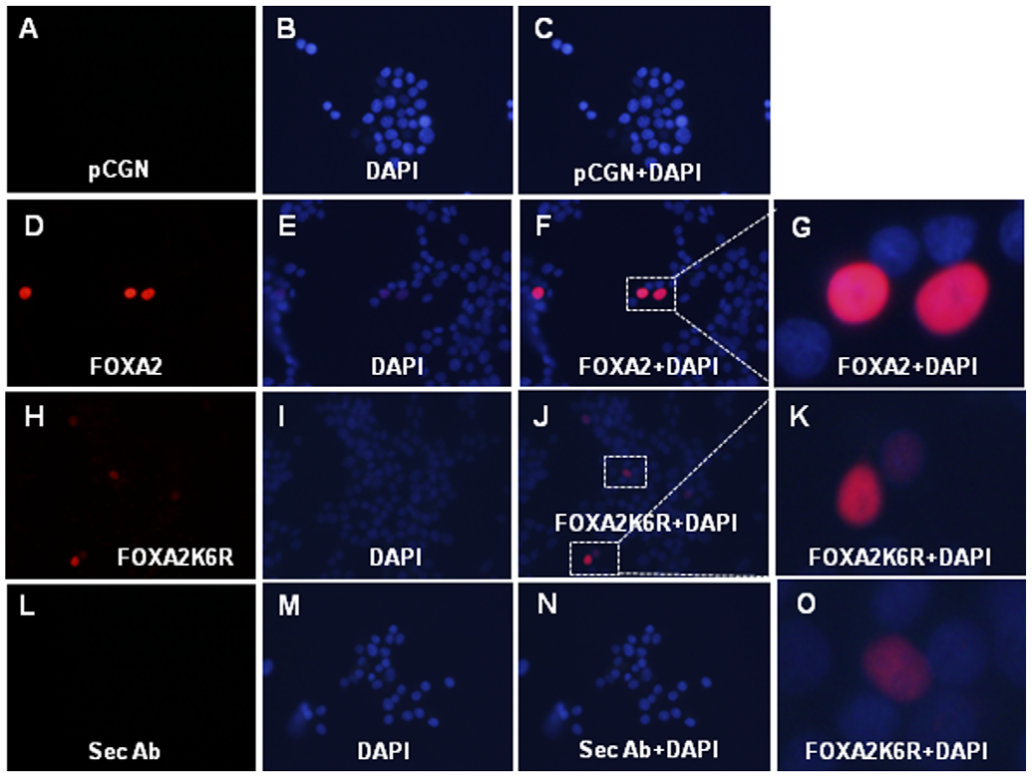
Nuclear localization of FOXA2 is independent of sumoylation. INS-1E cells plated on cover slips were not transfected (panels L,M,N) or transfected with pCGN empty vector (panels A,B,C) or HA epitope tagged FOXA2 (panels D,E,F,G) or FOXA2K6R mutant (panels H,I,J,K,O). Cells were fixed 36 hours post-transfection, permeabilized and stained with mouse HA antibody and fluorescent Alexa Fluor 594 donkey anti-mouse secondary antibody. No primary antibody was used for panels L,M,N. Cover slips were mounted in DAPI containing mounting media. Panels B,E,I and M are DAPI images. Panel C is superimposed A and B images. Panel F is superimposed D and E images. Panel J is superimposed H and I images. Panel N is superimposed L and M images. All images are at 400× magnification. Cells in the marked boxes in images F and J are digitally magnified and shown in panels G,K and O. Four times longer exposure were used for panels A, H and L. Two times lesser exposure was used for panel I.

SUMO modification influences subcellular localization of several substrate proteins. We examined if sumoylation is required for nuclear localization of FOXA2 by transfecting HA epitope tagged wild-type FOXA2 or sumoylation deficient FOXA2K6R or pCGN empty control vector into INS-1E cells and analyzed by immunofluorescent microscopy. Since FOXA2K6R protein is expressed at lower levels, 4 times longer exposure was required to detect FOXA2K6R protein. As shown in figure 7, both wild-type FOXA2 and sumoylation deficient FOXA2K6R proteins were localized in the nucleus suggesting that FOXA2 nuclear localization is independent of sumoylation.

### Sumoylation enhances FOXA2 transcriptional activity

**Figure 8:**
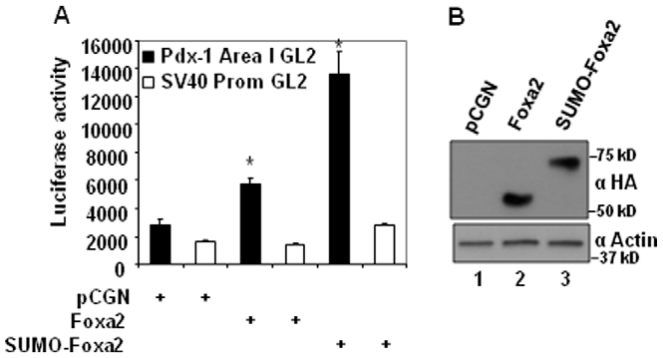
Sumoylation enhances FOXA2 transcriptional activity. Panel A. CV1 cells were transfected with 0.2 µg of Pdx-1 area I enhancer-SV40 early promoter driven luciferase reporter or SV40 early promoter driven luciferase control reporter along with 0.4 µg of expression vectors for Foxa2 or SUMO-1 fused Foxa2 or pCGN empty vector. Lysates were assayed for luciferase activity 36 hours post-transfection and normalized to total protein. Results from 3 experiments done in triplicates are shown as mean±SEM. * p<0.05 for Foxa2 transfected cells compared with pCGN transfected cells and SUMO-Foxa2 transfected cells compared with Foxa2 transfected cells. Panel B. Expression levels of FOXA2 and SUMO-FOXA2 detected by HA antibody is shown in the top panel and the internal standard actin detected by actin antibody is shown in the lower panel.

Sumoylation regulates transcriptional activities of target transcription factors. Since the K6 sumoylation site is present within the transcriptional activating domain IV/V of FOXA2, we examined if sumoylation alters FOXA2 transcriptional activity. *Pdx-1* area I region enhancer, a known target of FOXA2 [20], [34], linked to the SV40 minimal promoter-luciferase gene was used as a reporter. FOXA2 or constitutively sumoylated SUMO-FOXA2 or the pCGN empty vector was cotransfected with the *Pdx-1* area I-SV40 promoter-luciferase or the control SV40 promoter-luciferase reporters into CV1 cells. As shown in the figure 8A, FOXA2 activated the *Pdx-1* area I-SV40 promoter-luciferase approximately 2-fold, while constitutively sumoylated FOXA2 activated this enhancer 5-fold, indicating that sumoylation enhances FOXA2 transcriptional activity. Similar results were obtained when *Pdx-1* area I enhancer regulated TK minimal promoter-luciferase reporter was used instead of the *Pdx-1* area I-SV40 promoter-luciferase reporter (data not shown). Both FOXA2 and SUMO-FOXA2 were expressed at comparable levels indicating that the increased transcriptional activity was not a consequence of differences in the expression levels of FOXA2 and SUMO-FOXA2.

## Discussion

Reversible post-translational modification (PTM) of proteins is a common cellular strategy used for rapid modulation of protein activities and protein expression levels. PTMs regulate both the activity and expression levels of FOXA family members. While AKT and IKKα regulate FOXA2 activity by phosphorylating FOXA2 [22], [24], hepatocyte nuclear factor 6 (HNF6) that physically associates with FOXA2 and the acetyl transferase, CBP, regulate FOXA2 protein levels by stabilizing FOXA2 [29]. More recently Potter et al., showed that insulin-like growth factor I (IGF-1) regulates FOXA1 target gene expression by stabilizing FOXA1 protein [35].

Sumoylation is a PTM characterized by covalent attachment of small ubiquitin modifier peptide to cellular proteins [31]. The sumoylation pathway is involved in the regulation of various cellular processes such as, transcriptional activation/repression, DNA repair, chromatin dynamics, signal transduction, subcellular distribution of proteins, cell cycle regulation and apoptosis [30]. In this report we show that FOXA2 is sumoylated on K6 and K6 sumoylation is required for the stability of FOXA2 protein. Results of various experiments support these conclusions: (1) mutation of the SUMO acceptor lysine to arginine abolished sumoylation both *in vivo* and *in vitro*. (2) abolishing sumoylation by K6R substitution markedly reduced FOXA2 protein levels without affecting Foxa2 mRNA levels. (3) knocking down UBC9, the only SUMO conjugase essential for sumoylation, reduced FOXA2 protein levels. (4) SUMO-1 in-frame fusion conferred stability on unstable sumoylation deficient FOXA2K6R mutant protein. (5) SUMO ligase PIAS1 increased the steady state levels of FOXA2 by promoting FOXA2 sumoylation while a dominant negative SUMO ligase deficient PIAS1 mutant reduced both sumoylation and the steady state levels of non-sumoylated FOXA2.

From amongst more than 100 forkhead box family proteins, only FOXL2, FOXC1 and FOXC2 have been reported to be sumoylated [36]–[38]. Unlike FOXA2 in which sumoylation occurs on a single lysine residue located within a consensus sumoylation site, multiple lysine residues located at non-consensus sites were sumoylated in FOXL2. FOXL2 protein stability was enhanced by SUMO-1, the SUMO E2 conjugase UBC9 or the SUMO ligase, PIAS1 [38]. Interestingly, sumoylation deficient FOXL2 mutants exhibited steady state levels comparable to that of wild-type FOXL2 indicating that the stability of FOXL2 is not dependent on sumoylation site/s. This is in contrast to FOXA2 in which the K6 sumoylation site is essential for FOXA2 stability. These observations indicate that the sumoylation pathway regulates stability of forkhead box proteins both dependent and independent of direct sumoylation.

The SUMO moiety on sumoylated proteins provides an interface for interaction with proteins that contain a SUMO-interaction motif (SIM). A SIM is comprised of a core of hydrophobic residues often surrounded by acidic residues or phosphorylatable serines [39], [40]. FOXA2 interacts with various proteins including HNF6 and CBP, shown previously to stabilize FOXA2 protein [29]. LSDLL core sequence in HNF6, which resembles a SIM, was essential for synergistic interaction with FOXA2 [41]. Loss of such SUMO-SIM mediated interactions with other proteins that regulate FOXA2 stability could be responsible for destabilization and downregulation of sumoylation deficient FOXA2K6R protein.

Despite the importance of sumoylation for FOXA2 protein stability, only a small fraction of FoxA2 was sumoylated and the non-sumoylated FOXA2 appeared stable. This is in contrast to sumoylation deficient FOXA2K6R mutant which was inherently unstable. It has been observed for nearly all sumoylated proteins that a relatively small fraction of the available pool of a particular sumoylation substrate is sumoylated at steady state levels, yet the biological consequences elicited when sumoylation is abolished is huge and disproportionate to the amount of sumoylated substrate. This phenomenon termed “SUMO enigma” has been thought to be related to the dynamic nature of SUMO modification which is readily reversed by endogenous desumoylating activities [42]. According to the models proposed to explain “SUMO enigma”, sumoylation is required to confer competence to initiate a biological activity of a sumoylation substrate, for example, by incorporating the sumoylated substrate in to a transcriptional complex or localize to a particular cell compartment or subcompartment. Once the biological activity is initiated, desumoylation of previously sumoylated substrate does not affect already initiated biological activity. This model can be extended to explain the stability of non-sumoylated FOXA2 and the instability of non-sumoylatable FOXA2K6R mutant. Accordingly, both sumoylated FOXA2 and the non-sumoylated FOXA2 derived from desumoylation of previously sumoylated FOXA2 may remain stable whereas FOXA2K6R that is incapable of sumoylation may be destabilized.

Currently, the mechanisms involved in destabilization of sumoylation deficient FoxA2K6R are not clear. Previous studies have shown that preventing sumoylation by mutating the SUMO-acceptor lysine residue/s may expose ubiquitin-acceptor lysine residue/s located elsewhere in the protein, whose subsequent polyubiquitination will promote protein degradation. For example, K to R mutation that prevented sumoylation of the heterotrimeric G-protein regulator, phosducin, rendered the protein unstable by inducing polyubiquitination [43]. Similarly the stability of the paired-type homeodomain protein, Pax8, and the RNA helicases, p68 and p72, are reduced presumably because of polyubiquitination and proteasomal degradation when their sumoylation is prevented [44], [45]. However, inhibiting the ubiquitin-proteasomal degradation pathway marginally restored FOXA2K6R protein expression. This partial restoration was accompanied with an accumulation of low molecular weight peptides. Besides, high molecular weight ubiquitin-containing protein complexes characteristic of ubiquitinated degradation intermediates were detected in proteasome inhibitor treated FOXA2K6R transfected cells. These observations suggest that the K6R mutation may promote ubiquitin-modification of FOXA2K6R protein and the UPS along with additional proteases may be involved in FOXA2K6R degradation.

In summary, we have identified that FOXA2 is sumoylated on K6 and sumoylation regulates FOXA2 protein stability and transcriptional activity. FOXA2 plays a central role in the differentiation and functioning of pancreatic α and β cells [11], [13], [14], hepatocytes [16], [18], [46], and dopaminergic neurons [47], [48], whose dysfunction causes diseases such as, diabetes and Parkinson's disease. Considering the importance of sumoylation pathway for the stability and activity of FOXA2, sumoylation pathway may represent a potential target for drug development to treat such diseases.

## Supporting Information

Table S1 List and sequence of primers used for plasmid constructions.
(DOC)
