# Supplementary material for: Forkhead Box Protein A2 (FOXA2) Protein Stability and Activity Are Regulated by Sumoylation
Source: PLoS One. 2012 Oct 31;7(10):e48019. doi: 10.1371/journal.pone.0048019 (PMC3485284; doi:10.1371/journal.pone.0048019)
Supplement: Table S1 — List and sequence of primers used for plasmid constructions. (DOC) [file pone.0048019.s001.doc]

**Table S1. List and sequence of primers used for plasmid constructions**

FoxA2(T) 5’-gctctagaAGTATGCTGGGAGCCGTGAAGATG-3’

FoxA2(B) 5’-gcgggatccTTAGGATGAGTTCATAATAGGCCTGGAGT-3’

FoxA2K6R(T) 5’-gctctagaAGTATGCTGGGAGCCGTGAGGATGGAAG-3’

SUMO-1(T) 5’-tgctctagaATGTCTGACCAGGATAGCAGT-3’

SUMO-1∆GG,TAG(B) 5’-tgctctagaCGTTTGTTCCTGATAAACTTC-3’

SUMO-1GG∆TAG(B) 5’-tgctctagaACCCCCCGTTTGTTCCTGA-3’

Pdx-1 Area I(T) 5’-gcgacgcgtGCGTCTCTGTGAAGGGAAAGGGGGA-3’

Pdx-1 Area I(B) 5’-gcgacgcgtAGCTCCTGGTATCGTAAAATCGCCGA-3’

Nucleotides in lower case letters were designed into oligonucleotides to facilitate cloning and were not part of the indicated genes. Unerlined sequences correspond to the following restriction enzyme sites: BamHI in FoxA2(B) primer; MluI in Pdx-1 Area I(T), Pdx-1 Area I(B) primers; XbaI in FoxA2(T), FoxA2K6R(T), SUMO-1(T), and SUMO-1∆GG,TAG(B) primers. Abbreviation (T) and (B) in brackets denote the strand of the oligonucleotide.

**List and sequence of primers used for site directed mutagenesis**

FoxA2K256R(T) 5’-GAGCCCTTCCATCcTCACGGCTCCC-3’

FoxA2K365R(T) 5’-GGCCCACCTGAgGCCCGAGCACC-3’

Only the top strand sequence is shown for these mutagenic primers. The bottom strand is the complement of the top strand. Mutated nucleotides are shown in lower case.
